# Diagnostic value of ultrasound in children with transverse testicular ectopia

**DOI:** 10.3389/fped.2022.914139

**Published:** 2022-08-18

**Authors:** Wei Zhou, Shoulin Li, Hao Wang, Jianchun Yin, Xiaodong Liu, Junhai Jiang, Guanglun Zhou, Jianguo Wen

**Affiliations:** ^1^Pediatric Urodynamic Centre, Urology, The First Affiliated Hospital of Zhengzhou University, Zhengzhou, China; ^2^Department of Urology and Laboratory of Pelvic Floor Muscle Function, Shenzhen Children’s Hospital, Shenzhen, China

**Keywords:** ultrasound, children, transverse testicular ectopia, persistent Müllerian duct syndrome, diagnostic laparoscopy

## Abstract

**Objective:**

The study aimed to investigate the diagnostic value of ultrasound in children’s transverse testicular ectopia (TTE).

**Materials and methods:**

We retrospectively studies all TTE cases diagnosed in our hospital from January 2017 to December 2021. All cases were evaluated by ultrasound examination and compared to physical examination and diagnostic laparoscopy results.

**Results:**

This study included 14 TTE patients in total, with a median age was 1.08 years. In the 14 TTE, physical examination found 10 TTE cases, of which nine testes were located in the opposite scrotum, one testis was located in the opposite groin, and the other four testes were not observed by physical examination. All cases were diagnosed by preoperative ultrasound, and nine testes were located in the opposite scrotum, two testes were located in the opposite groin, and three testes were located next to the opposite iliac vessel in the abdominal cavity. Preoperative ultrasound showed the ectopic spermatic cord in six cases (6/14, 42.8%) and persistent Müllerian duct syndrome (PMDS) in one case (1/14, 7%). Diagnostic laparoscopy finally confirmed 14 cases of TTE, which was consistent with preoperative ultrasound, and the coincidence rate was 100% (14/14). Among the 14 cases of TTE, diagnostic laparoscopy showed that 12 cases had ectopic spermatic vessels and vas deferens (12/14, 85.7%), and six cases were associated with PMDS (6/14, 42.8%). When TTE was associated with the ectopic spermatic cord and PMDS, the diagnostic performance of diagnostic laparoscopy was better than that of preoperative ultrasound (*P* < 0.05). The testis volume of the affected side of TTE was less than that of the contralateral testis (*P* < 0.05).

**Conclusion:**

Ultrasonography is very helpful for the preoperative diagnosis of TTE in children, and it is suitable as a non-surgical method for locating ectopictestis. Preoperative assessment of the exact presence of PMDS is difficult and unclear. This may be related to factors such as pelvic developmental stages in infancy, examination techniques, and atypical imaging findings of PMDS.

## Introduction

Transverse testicular ectopia (TTE) is a rare disease in which both testes are located in the inguinal area or scrotum on the same side, each with independent blood vessels, epididymis, and vas deferens, and the scrotum on the other side is empty (1). TTE is subordinate to a special type of ectopic testis (ET) in cryptorchidism (2, 3).

Previous studies have suggested that the occurrence of TTE is related to persistent Müllerian duct syndrome (PMDS) (4–6). Children with TTE often present with a scrotal emptiness or a groin mass. In most previously reported cases, preoperative TTE was misdiagnosed as testicular atrophy, intra-abdominal testicle, or inguinal hernia (7). It is often discovered accidentally during operations such as cryptorchidism or inguinal hernia (8).

Our retrospective study aimed to explore ultrasonography in the ability to diagnose TTE in children.

## Materials and methods

Our study subjects were surgically confirmed TTE cases in our hospital from January 2017 to December 2021.

All ultrasound examinations were carried out by one of our teams of experienced pediatric urology sonographers. Patients were not sedated and were routinely placed in a supine position to fully expose the examination site. If necessary, a standing position and Valsalva maneuver examination were further performed. A high-resolution linear array transducer (MINDRAY DC-8) with a frequency of 5–12 MHz was used for all scans. Morphology and parenchymal homogeneity of the testes were performed on conventional grayscale images in three orthogonal planes. The low-velocity blood flow patterns of color Doppler flow imaging (CDFI) were used to detect the testicular parenchymal blood vessels. High color gain, low-flow filters, and low-velocity scales were used to optimize the detection of low-velocity arterial flow. If the testis could not be displayed in the scrotum and inguinal canal, the spermatic cord was tracked upward, along which the iliac fossa and retroperitoneum were included. After ultrasound showed both testes, the blood supply and direction of the testis and spermatic cord were observed. The position, shape, internal echo, size and volume, CDFI, and activity of both testes were measured and recorded, and the ultrasound images were stored in the PACS system for playback and analysis.

SPSS 26.0 software was used for statistical analysis (SPSS Inc., Chicago, IL, United States), measurement data such as testicular volume were expressed as mean ± standard deviation, and the *t*-test was used for comparison between groups. The enumeration data were expressed as rate (%), and the chi-square test was used for comparison between groups. A *P*-value of <0.05 was regarded as a statistically significant difference.

## Results

Our study involved 14 TTE in total during the period from January 2017 to December 2021. It included seven cases with left TTE and seven cases with right TTE. The ages of the patients ranged from 0.5 to 2.25 years, with a median of 1.08 years. The patients complained of scrotal emptiness in eight cases and groin mass in six cases. All cases underwent preoperative physical examination and ultrasonography, and the final diagnosis was confirmed by diagnostic laparoscopy. Chromosomal analysis of peripheral blood showed that all 14 cases were normal males with karyotype 46, XY.

In the 14 TTE, a physical examination found 10 TTE, of which nine testes were located in the opposite scrotum, one testis was located in the opposite groin, and the four other testes were not touched by physical examination. In addition, physical examination revealed six cases with inguinal hernia and two cases with scrotal hemangioma.

**FIGURE 1:**
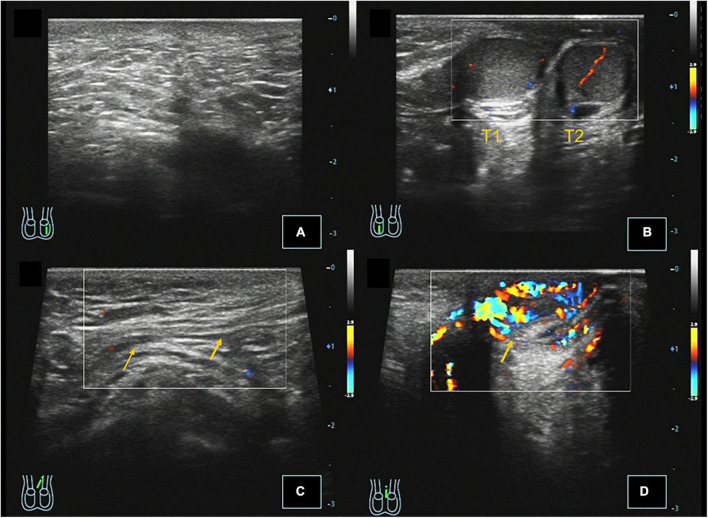
Case 3, 0.67 years, chief complaint: left scrotal emptiness. Preoperative ultrasound: **(A)** Longitudinal section of the left scrotum showing no testicular echo in the left scrotum. **(B)** Longitudinal section of the right scrotum showing that there were two testes echoes in the right scrotum, and CDFI showed that there were punctate blood flow signals in the testis parenchyma. **(C)** Longitudinal section of the left inguinal region showing that the left spermatic cord extends through the perineum to the right scrotum, and CDFI showed the punctate blood flow signal in the left spermatic cord. **(D)** Left scrotal longitudinal section showing hemiscrotal hemangioma, and CDFI showed that the blood flow signal is abundant in the mass.

**FIGURE 2:**
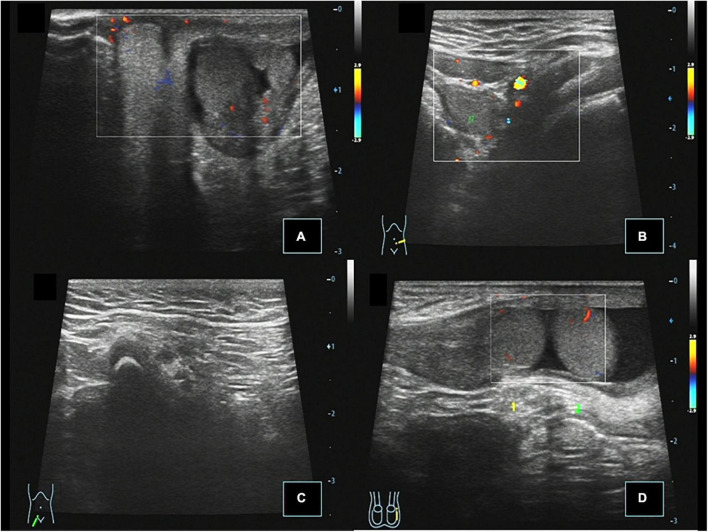
Case 14, 0.67 years, chief complaint: right scrotal emptiness. Preoperative ultrasound: **(A)** Cross section of the scrotum showing that the left testis is located in the left scrotum, and there was no testis echo in the right scrotum. **(B)** Cross section of the left lower abdomen showing that the right testis is located next to the left iliac vessel, and CDFI showed punctate blood flow signals in the testis parenchyma. **(C)** Longitudinal view of the right inguinal region showing no spermatic cord echo. **(D)** When the child was crying, the right testis passed through the left inner ring to the left inguinal canal.

All cases were diagnosed by preoperative ultrasound, and nine testes were located in the opposite scrotum, two testes were located in the opposite groin, and three testes were located next to the opposite iliac vessel in the abdominal cavity. Preoperative ultrasound showed the ectopic spermatic cord in six cases (6/14, 42.8%) and PMDS in one case (1/14, 7%). Preoperative ultrasound showed seven cases with inguinal hernia, three cases with contralateral cryptorchidism, and two cases with hemiscrotal hemangioma. Preoperative ultrasound showed normal testicular morphology and homogeneity in 14 cases of TTE, with no testicular atrophy, and CDFI showed blood flow signals in the testis parenchyma. The volume of the testis on the affected side of TTE measured by preoperative ultrasound was 0.33 ± 0.10 cm^3^, and the volume of the testis on the contralateral side was 0.37 ± 0.10 cm^3^. The volume of the testis on the affected side was smaller than that on the contralateral side, with a *t*-value of −2.910 and a *P*-value of <0.05, and the difference was statistically significant. The preoperative ultrasound features of transverse testicular ectopia in children were shown in Figures 1, 2.

Diagnostic laparoscopy finally confirmed 14 cases of TTE, which was consistent with preoperative ultrasound, and the coincidence rate was 100% (14/14). Among the 14 cases of TTE, diagnostic laparoscopy showed that 12 cases had ectopic spermatic vessels and vas deferens (12/14, 85.7%), and six cases were associated with PMDS (6/14, 42.8%). Among the six cases of PMDS, three cases showed dysplastic uterine structures in the pelvis and three cases showed fallopian tube umbrella structures adjacent to the bilateral gonads. In addition, diagnostic laparoscopy showed seven cases of TTE with inguinal hernia and three cases of TTE with contralateral cryptorchidism. The demographic details of the 14 cases of transverse testicular ectopia in children are shown in Table 1.

**TABLE 1:** Demographics of 14 cases of transverse testicular ectopia in children.

Information	Number of cases	Percentage of total cases
The age of surgery in children with TTE was within 1 year old	10	71.43%(10/14)
The age of surgery in children with TTE was older than 1 year old	4	28.57%(4/14)
TTE on the left side	7	50.00%(7/14)
TTE on the right side	7	50.00%(7/14)
TTE associated with contralateral cryptorchidism	3	21.43%(3/14)
TTE had ectopic spermatic vessels and vas deferens	12	85.71%(12/14)
TTE had normal spermatic vessels and vas deferens	2	14.29%(12/14)
TTE associated with PMDS	6	42.86%(6/14)
TTE associated with inguinal hernia	7	50.00%(7/14)
TTE associated with scrotal hemangioma	2	14.29%(2/14)

The preoperative diagnostic performance of ultrasonography was significantly better than that of physical examination, the Pearson chi-square value was 4.667, *P*-value was <0.05, and the difference was statistically significant. The results of preoperative ultrasonography were fully consistent with diagnostic laparoscopy, and the diagnostic coincidence rate of preoperative ultrasonography in children with TTE was 100% (14/14). When TTE was associated with the ectopic spermatic cord and PMDS, the diagnostic performance of diagnostic laparoscopy was better than that of preoperative ultrasound, the Pearson chi-square values were 5.600 and 3.914, *P*-value of <0.05, and the difference was statistically significant. The comparison of diagnostic accuracy of preoperative ultrasound and diagnostic laparoscopy in children with TTE associated with PMDS and ectopic spermatic cord is shown in Table 2.

**TABLE 2:** Comparison of diagnostic accuracy of preoperative ultrasound and diagnostic laparoscopy in children with transverse testicular ectopia (TTE) associated with persistent Müllerian duct syndrome (PMDS) and ectopic spermatic cord [cases (%)].

Diagnostic methods	Total cases	TTE associated with PMDS	TTE associated with ectopic spermatic cord
Preoperative ultrasound	14	1 (7%)	6 (42.8%)
Diagnostic laparoscopy	14	6 (42.8%)	12 (85.7%)
Pearson Chi-square value (χ^2^)		3.914	5.600
*P*-value		0.001	0.001

Among the 14 cases of TTE, 12 cases of TTE with ectopic spermatic vessels and vas deferens were performed laparoscopy-assisted transscrotal mediastinal orchiopexy (Ombredanne operation), and the remaining two cases of TTE with normal spermatic vessels and vas deferens were performed laparoscopic orchidopexy. The laparoscopic hernia repair was performed for seven cases of TTE associated with inguinal hernia, and inguinal orchidopexy was performed for three cases of TTE associated with contralateral cryptorchidism. Bilateral gonad and surrounding tissue biopsies were performed for six cases of TTE associated with PMDS. Postoperative histologic and pathological reports showed dysplastic seminiferous tubules in six cases of bilateral gonads, and fallopian tube structures in the surrounding tissues of gonads in three cases. The age, clinical manifestations, and management of 14 cases of transverse testicular ectopia in children are shown in Table 3.

**TABLE 3:** Age, clinical manifestations, and management of 14 cases of transverse testicular ectopia in children.

Serial number	Age (years)	Affected side	Preoperative ultrasound	Diagnostic laparoscopy (testis location)	Surgical procedure	Histological diagnosis
1	0.75	L	L TTE	L TTE associated with PMDS (R inguinal canal, ESC), R IH	TMO (OP) + HR + GB	Bilateral gonads: dysplastic testes (with visible seminiferous tubule structures inside)
2	0.75	R	R TTE	R TTE (L inguinal canal, ESC)	TMO (OP)	–
3	0.67	L	L TTE	L TTE (R scrotum), L SH	LO	–
4	2.25	R	R TTE	R TTE associated with PMDS (L deep inguinal ring, ESC), L IH	TMO (OP) + HR + GB	Bilateral gonadal parenchyma: dysplastic testes (with visible seminiferous tubules inside) Bilateral gonadal appendages: fallopian tube-like structures
5	1.25	R	R TTE	R TTE associated with PMDS (L inguinal canal, ESC), L IH	TMO (OP) + HR + GB	Bilateral gonadal parenchyma: dysplastic testes (with visible seminiferous tubules inside) Bilateral gonadal appendages: fallopian tube-like structures
6	0.67	L	L TTE	L TTE (R inguinal canal, ESC), R IH	TMO (OP) + HR	–
7	0.83	L	L TTE	L TTE associated with PMDS (R inguinal canal, ESC), R IH	TMO (OP) + HR + GB	Bilateral gonadal parenchyma: dysplastic testes (with visible seminiferous tubules inside) Left gonadal appendages: fallopian tube-like structures
8	0.75	R	R TTE	R TTE (L inguinal canal, ESC), L IH	TMO (OP) + HR	–
9	0.83	L	L TTE	L TTE (R scrotum), L SH	LO	–
10	2.08	B/L	L TTE, R EAT	L TTE associated with PMDS (R inguinal canal, ESC), R EAT	TMO (OP) + GB	Bilateral gonads: dysplastic testes (with visible seminiferous tubule structures inside)
11	1.00	L	L TTE	L TTE (R inguinal canal, ESC), R IH	TMO (OP) + HR	–
12	0.50	B/L	L EAT and R TTE	L EAT, R TTE associated with PMDS (left iliac fossa, ESC)	TMO (OP) + GB	Bilateral gonads: dysplastic testes (with visible seminiferous tubule structures inside)
13	2.08	B/L	L EAT and R TTE	L EAT, R TTE (left iliac fossa, ESC)	TMO (OP)	–
14	0.67	R	R TTE	R TTE (left iliac fossa, ESC)	TMO (OP)	–

L, left; R, right; B/L, bilateral; EAT, extra-abdominal testis; TTE, transverse testicular ectopia; PMDS, persistent Müllerian duct syndrome; IH, inguinal hernia; ESC, ectopic spermatic cord; SH, scrotal hemangioma; LO, laparoscopic orchiopexy; TMO, transscrotal mediastinal orchiopexy; OP, Ombredanne’s procedure; GB, gonadal biopsy; HR, hernia repair.

**FIGURE 3:**
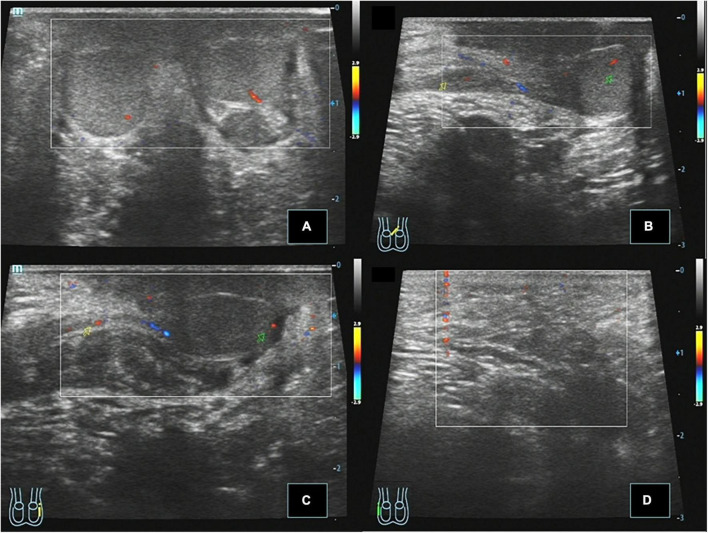
Case 14, 0.8 years, 1 month after Ombredanne operation. Postoperative ultrasound: **(A)** Longitudinal section of the scrotum showing that both testes were located in the bilateral scrotum, respectively, and CDFI showed that the blood flow signal was normal in the parenchyma. **(B)** Longitudinal section of the left testis showing that the left spermatic cord continues to the left testis through the left inguinal region. **(C)** Longitudinal view of the left inguinal region showing that the right spermatic cord echoes across the perineum and continues with the testis in the right scrotum. **(D)** Longitudinal view of the right inguinal region showing no spermatic cord echo.

All 14 cases of TTE were followed up by ultrasound, and the follow-up period was 2 months to 5 years. Postoperative ultrasound of the 14 cases of TTE showed that the testes were located in the scrotum with normal morphology (Figure 3). The CDFI showed punctate blood flow signals in the testicular parenchyma. Displacement, atrophy, and malignant transformation of the reset testis were not observed.

## Discussion

The pathogenesis of TTE is still unclear. Previous studies have suggested that there are five tails of the gubernaculum testis during testicular development, which are attached to the perineum, the front of the pubis, Scarpa’s triangle in the thigh, the region of the inguinal ligament medial to the anterior superior iliac spine, and the bottom of the scrotum. Normally, the testis descends along the guide belt that attaches to the bottom of the scrotum and terminates in the scrotum. When other tails of the gubernaculum replace the tail at the bottom of the scrotum to guide the descending of the testis, the testicular gubernaculum terminates outside the scrotum, and the testis is ectopic (9, 10). Based on this theory, ET can be divided into four types, namely, superficial inguinal, perineal, penile, and transverse testicular ectopia. Some scholars believe that the occurrence of TTE is related to that of both testes originating from the same genital ridge. Some other scholars believe that the mesonephric ducts adhere or fuse during embryonic development, the bilateral testes and spermatic cords tightly adhere, and the descending of one testis drags the other testis down at the same time, forming a testis transverse ectopic (11).

Persistent Müllerian duct syndrome is a very rare pathological association with TTE. PMDS is a rare male pseudohermaphroditism, which refers to the continuous development of Müllerian ducts due to the lack of Müllerian inhibiting factor (MIF) or anti-Müllerian hormone (AMH) during embryonic development in male individuals with normal genotypes, and further differentiates into the uterus, fallopian tubes, and upper vagina (5). In children with PMDS, the lack of MIF can lead to obstruction of testicular descent. When one testis descends, it drags the remnants of the Müllerian duct and the other testis into the same inguinal canal, resulting in an inguinal hernia and testicular ectopia (6, 12). Among the 14 TTE cases in this group, six cases of TTE were associated with PMDS, and the chromosomal analysis of peripheral blood revealed that all the cases were normal males with karyotype 46, XY.

Most patients with TTE complained of scrotal emptiness or groin mass. Among the 14 cases in this group, eight cases complained of scrotal emptiness and six cases complained of groin mass. Using the Gauderer method, TTE can be divided into three types according to the accompanying lesions: type I, TTE with an inguinal hernia; type II, TTE with PMDS or primordial uterus; and type III: TTE with other abnormalities, such as hydrocele, suburethral cleft, epididymal malformation, scrotal anomaly, horseshoe kidney, and ureteropelvic junction obstruction (13). Other literature reports classify TTE into three types according to different locations of the testis: inguinal, intra-abdominal, and scrotal (14). We realize that some TTE cases may have more than two kinds of combined lesions, and the classification may be repeated according to the Gauderer method. The classification according to the location of the ectopic testis may be helpful for the selection of surgical methods.

When the testis is difficult to touch on physical examination, other non-operative diagnostic modalities can help determine the location of the impalpable testis, such as ultrasound (US), computed tomography (CT), emission computed tomography (ECT), magnetic resonance imaging (MRI), magnetic resonance venography (MRV), and arteriography (15). CT, ECT, and MRI can show cord-like structures spanning from one side of the abdominal cavity to the other side, and it is possible to observe whether the testis is located around the iliac vessels or behind the bladder. However, CT and ECT have large ionizing radiation, and MRI is expensive, arteriography and venography require general anesthesia, which are invasive and difficult to be widely used in infants and young children (16, 17).

Ultrasound is the most widely used imaging method in pediatric urology. It has the advantages of non-invasive, economical, non-radioactive, fast, easy, and repeatable dynamic observations (18–20). Preoperative ultrasound can evaluate not only the location, size, shape, and blood supply of the testis but also comorbidities such as inguinal hernia, scrotal hemangioma, and contralateral cryptorchidism at the same time (21). In our clinical experience, all children with non-palpable testes (NPTs) were evaluated preoperatively with ultrasound. Preoperative ultrasound can provide valuable information for the diagnosis and treatment of NPT in children, including the classification of NPT, the anatomical location of the testis, and the degree of testicular activity. This is important as knowledge of the anatomical location and range of motion of the non-palpable testes prior to intervention can improve surgical performance and minimize the risk of complications.

Among the 14 cases of TTE in this group, testis in 10 cases of TTE was palpable by preoperative physical examination, and 14 cases were diagnosed by preoperative ultrasound. The statistical analysis showed that the diagnostic accuracy of preoperative ultrasound in children with TTE was significantly better than that of the preoperative physical examination (*P* < 0.05). Preoperative ultrasonography and diagnostic laparoscopy were fully consistent in the diagnosis of TTE in children, and the diagnostic coincidence rate was 100%. The statistical results of this study show that ultrasound has incomparable advantages in the preoperative diagnosis of TTE in children. The preoperative ultrasonographic features of our 14 TTE cases were as follows:

In the 14 cases of TTE, nine testes were located in the opposite scrotum, two testes were located on the opposite groin, and three testes were located next to the opposite iliac vessel in the abdominal cavity. Ultrasound showed no testis echoes on one side of the scrotum and groin, while two testes echoes were observed in the contralateral scrotum, groin, or abdominal cavity. Previous studies have reported that TTE was more common in the contralateral scrotum, and our results are consistent with previous case reports (4).

The testis in 14 cases with TTE was oval in shape, with uniform internal echo, which was consistent with normal testis. The volume of the testis on the affected side and contralateral side of TTE measured by ultrasound were 0.33 ± 0.10 and 0.37 ± 0.10 cm^3^. The volume of the affected side testis was smaller than that of the contralateral testis (*P* < 0.05), which was consistent with the results of previous studies (5). When the testis was associated with by atrophy, torsion, and malignant transformation, the intraparenchymal echo may be inhomogeneous, showing flaky hypoechoic and liquefied anechoic areas (18–20).

The transverse ectopic testis and normal testis are relatively independent, and each testis has its own spermatic cord and epididymis connected to it. The blood flow signal across the parenchyma of the ectopic testis is similar to that of the normal testis, and the blood supply of both testes originates from the arteries in the respective spermatic cords. The arterial blood flow velocity curve in the testis parenchyma showed low velocity and low resistance. Limited by the size and developmental stage of the testis, the testicular parenchyma usually shows punctate blood flow in the prepubertal period. After puberty, subcapsular artery and centripetal artery blood flow signals can be seen in the testicular parenchyma.

The ultrasonographic findings of PMDS revealed a cord-like hypoechoic mass or a cystic anechoic mass between the bladder and rectum in the pelvis. CDFI showed no obvious blood flow signal in the mass. Among the 14 cases of TTE in this group, one case of TTE associated with PMDS was found by preoperative ultrasound, while six cases of TTE associated with PMDS were confirmed by diagnostic laparoscopy. When TTE was associated with PMDS, the diagnostic performance of diagnostic laparoscopy was better than that of preoperative ultrasound (*P* < 0.05). In most of the previously reported cases, PMDS was often diagnosed accidentally during operations such as cryptorchidism or inguinal hernia (21), and correct diagnosis could not be made preoperatively. We analyzed that the reasons for the missed diagnosis of PMDS by preoperative ultrasound may be related to the following factors: Sonographers’ lack of the theoretical knowledge of TTE associated with PMDS; the examination ended when the scans of both testes and spermatic cords, ignoring further examination of the pelvis; and the uterus is a deep pelvic organ, and infants and young children need to hold back a lot of urine before ultrasound examination of the uterus and ovaries. Children with TTE performed ultrasound for scrotal emptiness, which shows a lack of preparation for holding back a lot of urine, resulting in the limited acoustic window display of deep pelvic organs.

Among the 14 cases of TTE, seven cases of TTE were associated with inguinal hernia, and ultrasonography showed that the abdominal contents (bowel and omental tissue echoes) bulged downward into the inguinal canal. A total of two cases of TTE were associated with contralateral scrotal hemangioma, and ultrasound showed hypoechoic masses in the epidermis and subcutaneous soft tissue of the contralateral scrotum, the internal echo was not uniform, CDFI showed rich blood flow signal in the mass. In previously reported cases, there were few descriptions of TTE associated with scrotal hemangioma (1, 22, 23). Among the 14 cases of TTE in this group, only two cases of TTE had no ectopic spermatic cord; two cases of TTE were associated with hemiscrotal hemangioma, and the testes were ectopic to the contralateral scrotum. We further speculate that hemiscrotal hemangioma may cause the ectopic testis to traverse into the contralateral scrotum, which needs to be further verified in a large sample in the future. On the other hand, diagnostic laparoscopy confirmed the presence of the ectopic spermatic cord in 12 cases of TTE, but preoperative ultrasound only showed the ectopic spermatic cord in six cases of TTE. The statistical analysis results showed that the diagnostic performance of diagnostic laparoscopy was better than that of preoperative ultrasound when TTE was associated with the ectopic spermatic cord in children, suggesting that it was difficult to find the course of the ectopic spermatic cord by using non-surgical diagnostic methods such as ultrasound.

The surgical methods for TTE include transscrotal mediastinal orchiopexy (Ombredanne operation), orchiopexy, extraperitoneal orchiopexy, bilateral testes fixed in the same hemiscrotum, Fowler-Stephens orchiopexy, testicular transplantation surgery, and orchiectomy (5, 7). Transscrotal mediastinal orchiopexy is the most commonly used procedure for TTE. It refers to the descent of the ectopic testis and the normal testis together through the inguinal canal on the same side of the scrotum, and then the ectopic testis is fixed in the contralateral scrotum through the scrotal mediastinum. Transscrotal mediastinal orchiopexy is suitable for TTE with sufficient spermatic cord length but bilateral spermatic cord fusion and difficult separation. When the bilateral spermatic cord and vas deferens are not easy to separate and the spermatic cord is only long enough to be fixed in the upper part of the ipsilateral scrotum, we can choose to fix both testicles in the same hemi scrotum (8, 9). Fortunately, after the spermatic cord and vas deferens were fully separated, 12 children of TTE with the ectopic spermatic cord in this group were performed laparoscopic-assisted transscrotal mediastinal orchiopexy, and the remaining two children of TTE with a normal spermatic cord were performed laparoscopic orchiopexy. In this group of cases, the Fowler-Stephens orchiopexy and other procedures were not used. The postoperative ultrasound follow-up showed that the reduced tests of the 14 TTE children were all fixed at the bottom of the scrotum, with a normal shape; the CDFI showed punctate blood flow signals in the testicular parenchyma; and testicular atrophy, malignant transformation, testicular epididymitis were not found.

With the development of minimally invasive surgical techniques, laparoscopy can assess not only the blood supply and development of bilateral testes, spermatic cords, and vas deferens under direct vision but also the length of spermatic cords and vas deferens and the presence or absence of Müllerian duct residues at the same time (11, 12). Laparoscopic assistance cannot only free the spermatic cord and vas deferens fully but also treat the unregenerate Müllerian ducts while performing testicular fixation. It also has the advantages of less damage to the spermatic vessels and vas deferens, faster postoperative recovery, and lower infection rate. However, it is still inconclusive whether the Müllerian duct is fully removed under laparoscopy during the operation. Although the residual Müllerian duct may have the risk of long-term malignant transformation, complete removal of the Müllerian duct will affect the blood supply of the testis and the vas deferens and the development of the testis. Therefore, complete resection should be considered only when the Müllerian duct affects the reduction of testicular descent (13, 14). In this group, there were six cases of TTE associated with PMDS, which were all successfully performed Ombredanne’s procedure after fully dissociating spermatic cord vessels and vas deferens by laparoscopy. Postoperative histologic examination showed that the gonads in six cases of TTE associated with PMDS were dysplastic testis, and the residual Müllerian ducts around the gonads in three cases were fallopian tube-like structures. Histologic findings were consistent with the disease characteristics of TTE associated with PMDS.

We recognize the limitations of our study: the study had a small sample size and lack of a normal control group for comparison, and there was no randomization of cases. In addition, limited by the stage of testicular development in infancy, the size and volume of the testis were small, the follow-up time was short, and the results of testicular development assessment in adulthood were lacking. These deficiencies need to be improved by designing more scientifically reasonable prospective studies and long-term follow-ups.

## Conclusion

Ultrasonography is very helpful for the preoperative diagnosis of TTE in children, and it is suitable as a non-surgical method for locating ectopic testis. Preoperative assessment of the exact presence of PMDS is difficult and unclear. This may be related to factors such as pelvic developmental stages in infancy, examination techniques, and atypical imaging findings of PMDS.

## Data availability statement

The original contributions presented in this study are included in the article/supplementary material, further inquiries can be directed to the corresponding author.

## Ethics statement

The studies involving human participants were reviewed and approved by the Ethics Committee of Shenzhen Children’s Hospital. Written informed consent to participate in this study was provided by the participants’ legal guardian/next of kin.

## Author contributions

WZ designed the study, analyzed the data, and wrote the manuscript. JW and SL funded the project. WZ, HW, JY, JJ, and GZ confirmed all the data in the manuscript. All authors have read and approved the final manuscript.
